# “Idiopathic” minimal change nephrotic syndrome: a podocyte mystery nears the end

**DOI:** 10.1152/ajprenal.00219.2023

**Published:** 2023-10-05

**Authors:** Sumant S. Chugh, Lionel C. Clement

**Affiliations:** Glomerular Disease Therapeutics Laboratory, Department of Internal Medicine, Rush University Medical Center, Chicago, Illinois, United States

**Keywords:** atopy, cytokine storm, minimal change disease, nephrotic syndrome, podocyte

## Abstract

The discovery of zinc fingers and homeoboxes (ZHX) transcriptional factors and the upregulation of hyposialylated angiopoietin-like 4 (ANGPTL4) in podocytes have been crucial in explaining the cardinal manifestations of human minimal change nephrotic syndrome (MCNS). Recently, uncovered genomic defects upstream of *ZHX2* induce a *ZHX2* hypomorph state that makes podocytes inherently susceptible to mild cytokine storms resulting from a common cold. In *ZHX2* hypomorph podocytes, ZHX proteins are redistributed away from normal transmembrane partners like aminopeptidase A (APA) toward alternative binding partners like IL-4Rα. During disease relapse, high plasma soluble IL-4Rα (sIL-4Rα) associated with chronic atopy complements the cytokine milieu of a common cold to displace ZHX1 from podocyte transmembrane IL-4Rα toward the podocyte nucleus. Nuclear ZHX1 induces severe upregulation of *ANGPTL4*, resulting in incomplete sialylation of part of the ANGPTL4 protein, secretion of hyposialylated ANGPTL4, and hyposialylation-related injury in the glomerulus. This pattern of injury induces many of the classic manifestations of human minimal change disease (MCD), including massive and selective proteinuria, podocyte foot process effacement, and loss of glomerular basement membrane charge. Administration of glucocorticoids reduces *ANGPTL4* upregulation, which reduces hyposialylation injury to improve the clinical phenotype. Improving sialylation of podocyte-secreted ANGPTL4 also reduces proteinuria and improves experimental MCD. Neutralizing circulating TNF-α, IL-6, or sIL-4Rα after the induction of the cytokine storm in *Zhx2* hypomorph mice reduces albuminuria, suggesting potential new therapeutic targets for clinical trials to prevent MCD relapse. These studies collectively lay to rest prior suggestions of a role of single cytokines or soluble proteins in triggering MCD relapse.

## Graphical abstract

**Figure:**
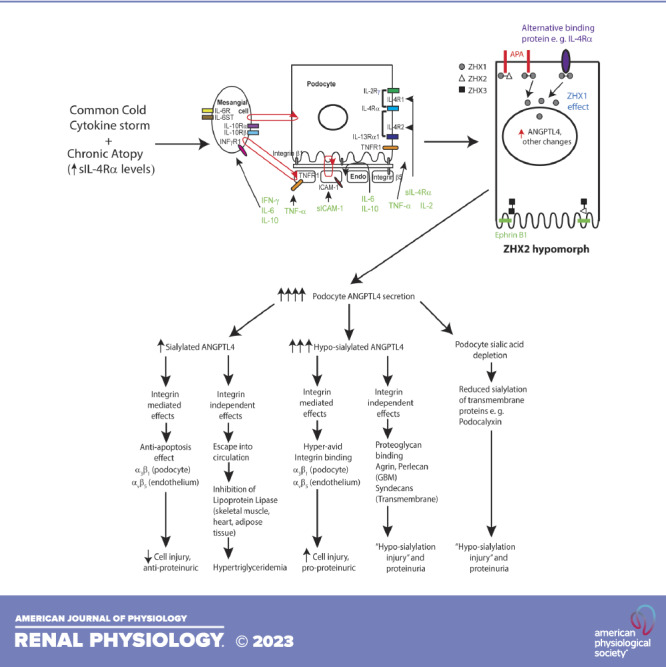

## INTRODUCTION

Minimal change nephrotic syndrome (MCNS), more frequently referred to as minimal change disease (MCD), is a major cause of nephrotic syndrome in children and a less frequent cause in adults (1). Below the age of 6 yr, it is several-fold more common than focal and segmental glomerulosclerosis (FSGS). MCD has several unique features, including the acute onset of edema (often overnight), urine profile showing predominant albuminuria, a history of atopy in nearly half of all patients, and frequent relapses often triggered by a common cold. In kidney biopsies, foam cells are variably noted; there is a lack of inflammation, complement, or immunoglobulin deposition; glomeruli appear normal on light microscopy; tubulointerstitial fibrosis is absent despite heavy proteinuria; and electron microscopy reveals diffuse effacement of podocyte foot processes (2). Patients often respond to glucocorticoids alone initially and sometimes need cytotoxic medications to prevent frequent relapses. Other than the toxicity of cytotoxic therapy, it is infrequent for patients to progress to chronic kidney disease. Some interesting aspects of this disease that have allowed us to determine MCD pathogenesis are the frequent history of atopy (3) and trigger of relapse by a common cold (4). Others, like the acute onset of edema, remain under investigation. Also, the absence of chronic kidney disease despite heavy proteinuria is being used by us to identify potential therapeutic targets to treat chronic kidney disease caused by more common disorders like diabetic nephropathy (5).

Numerous pathways are involved in any disease, some central and others peripheral to disease pathogenesis. Central pathogenetic pathways explain the majority of unique clinical and pathological features. For this reason, this review will discuss the zinc fingers and homeoboxes proteins and angiopoietin-like 4 (ZHX-ANGPTL4) pathway in detail (Table 1) and then highlight numerous peripheral and incompletely investigated pathways.

**Table 1.** Specific location of MCD mechanisms and mechanistic basis of its clinical and pathological features in ZHX-ANGPTL4 pathway publications

Observation	Mediators	Reference	Figures and Tables
Upstream pathways that trigger MCD			
Common cold cocktails that induce MCD relapse	Cytokines and soluble receptors	6	Fig. 1, *A* and *C–G*; Supplemental Fig. S1*B*
Mechanisms of common cold-induced MCD relapse	Cytokines and soluble receptors	6	Fig. 8, *A*, *C*, *E*, *F*, and *H*
Cytokine depletion to prevent common cold MCD relapse	Antibodies against cytokines, soluble receptors	6	Fig. 3*A*
Cell membrane to nuclear migration of ZHX proteins			
Podocyte ZHX protein expression		7	Figs. 2*A* and 8, *C* and *G*
Tubular ZHX protein expression		8	Supplemental Fig. S3*A*
APA, ephrin B1 association with ZHX proteins in podocytes		8	Figs. 4, 5, and 6, *B*–*D*; Supplemental Fig. S3, *C*–*E*
ZHX2 hypomorph state in MCD		8	Fig. 1*B*
Human genomic basis of *ZHX2* hypomorph state in MCD		6	Fig. 6; Table 4; Supplemental Figs. S6 and S7
ZHX2 hypomorph state in rodents		8	Fig. 2
ZHX2 hypermorph state in rodents		8	Fig. 3
Podocyte gene regulation by ZHX proteins		7	Table 2
*Angptl4, ANGPTL4* regulation by ZHX proteins		8	Fig. 6*F*; Supplemental Fig. S6
*Angptl4* upregulation by anti-APA antibodies	ZHX1	8	Fig. 6, *A*–*C* and *E*
Consequences of podocyte ANGPTL4 upregulation in MCD			
ANGPTL4 in human MCD (glomeruli, blood, urine)		9	Supplemental Figs. S9 and S10
ANGPTL4 sialylation and hyposialylation		9	Fig. 4; Supplemental Figs. S6*A* and S7
ANGPTL4-integrin interactions	ANGPTL4, ITGB5	10	Supplemental Fig. S5, *D* and *E*
Increased cellular injury with hyposialylated ANGPTL4	Integrin and nonintegrin mediated	10	Supplemental Fig. S5*C*
Diffuse podocyte foot process effacement	Hyposialylated ANGPTL4	9	Fig. 2, *C*, *I* and *J*; Supplemental Fig. S5*D*
Loss of GBM charge	Hyposialylated ANGPTL4	9	Supplemental Fig. S5
Selective proteinuria	Hyposialylated ANGPTL4	9	Fig. 3*D*
Hypertriglyceridemia in MCD	Sialylated ANGPTL4	10	Figs. 1, *A* and *E*, 2, *C*–*G*, and 6, *A* and *B*
Initiation of hypercholesterolemia (using FSGS models)	CCD-secreted PCSK9 (chaperone for ENaC)	11	Figs. 1 –6
Glucocorticoid sensitivity of *Angptl4* (in vivo)		9	Fig. 3*G*; Supplemental Fig. S6*A*
Sialylation-based therapeutics		9	Fig. 4, *E*–*G*; Supplemental Fig. S8, *A* and *B*
Cellular protective effects of sialylated ANGPTL4	Integrin and nonintegrin mediated	10	Supplemental Fig. S5*C*

ANGPTL4, angiopoietin-like 4; APA, aminopeptidase A; FSGS, focal and segmental glomerulosclerosis; GBM, glomerular basement membrane; MCD, minimal change disease; ZHX, zinc fingers and homeoboxes; ITGB5, integrin-β5; CCD, cortical collecting duct; PCSK9, proprotein convertase subtilisin/kexin type 9; ENaC, epithelial Na^+^ channel.

## ZHX-ANGPTL4 PATHWAY

### Scientific Origin of the ZHX-ANGPTL4-Related Pathway

In the late 1990s, we characterized the immunoreactivity of the γ-2 fraction of a sheep anti-rat whole glomerular nephrotoxic serum (NTS), which induces massive complement- and leukocyte-independent heterologous phase proteinuria in rats (12). The most prominent reactive protein identified on Western blot was aminopeptidase A (APA, product of the *Enpep* gene) that had previously been identified by others (13) as a nephritogenic protein. In the previous study (13), monoclonal anti-APA antibodies bound to the podocyte surface and induced massive albuminuria in BALB/c mice (on retrospective review by the authors, mice were purchased from Jackson laboratories, so these were BALB/cJ mice). In the early 2000s, discovery phase studies using whole glomerular RNA for differential gene expression were conducted in γ-2-NTS-injected Sprague-Dawley rats. Of the nearly 40 differentially expressed genes noted, an unknown downregulated gene, subsequently cloned and identified as transcriptional factor zinc finger and homeoboxes 3 (*Zhx3*) (7), and a highly upregulated gene, angiopoietin-like 4 (*Angptl4*) (9), previously known to be involved in pathogenesis of hypertriglyceridemia, were investigated. Over the past two decades, all stages of this initial discovery have been integrated by showing that all three ZHX proteins regulate podocyte *Angptl4* expression, and anti-APA antibodies injected into *Zhx2*-deficient BALB/cJ mice induce podocyte *Angptl4* upregulation and severe albuminuria (8).

### ZHX Proteins and MCD

**Figure 1.**
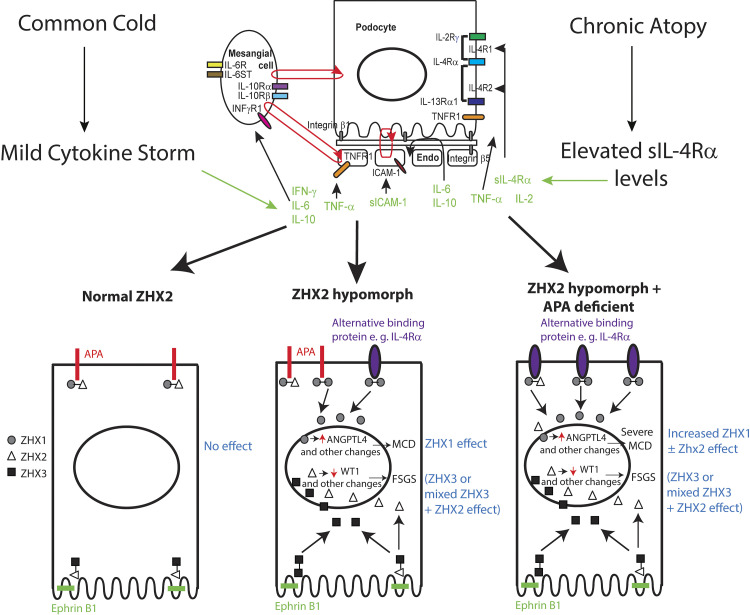
Schematic representation of minimal change disease (MCD) relapse induced by a common cold cytokine storm in the setting of chronic atopy and a *Zhx2* hypomorph state. *Top*: cytokine cocktail components, relevant cytokine receptors in podocytes, endothelial and mesangial cells, and the contribution of sIL-4Rα by chronic atopy. IL-4Rα forms two receptor complexes, IL-4R1 and IL-4R2, in podocytes. *Bottom*: normal zinc fingers and homeoboxes (ZHX) protein heterodimer distribution in in vivo podocytes (*left*), altered ZHX protein heterodimer and homodimer distribution in the *Zhx2* hypomorph state (*middle*), and dual *Zhx2* hypomorph and *Enpep*-deficient states (*right*). The normal transmembrane binding partner for ZHX3-ZHX2 at the slit diaphragm is ephrin B1 and for ZHX1-ZHX2 in the podocyte body is aminopeptidase A (APA). In a *Zhx2*-deficient state, ZHX1 homodimers and possibly ZHX1-ZHX2 heterodimers also bind to the cytoplasmic tail of IL-4Rα. In a dual *Zhx2* hypomorph-*Enpep*-deficient state, a larger amount of ZHX1 homodimers and ZHX1-ZHX2 heterodimers likely bind IL-4Rα. ZHX-mediated MCD relapse pathways start from the podocyte body, whereas ZHX-mediated focal and segmental glomerulosclerosis (FSGS) relapses originate from the slit diaphragm and foot processes. Endo, endothelium; ICAM-1, intercellular adhesion molecule-1; sICAM-1, soluble ICAM-1; TNFR1, tumor necrosis ractor receptor type 1; IL-2R γ, interleukin 2 receptor subunit γ; IL-4R α, interleukin 4 receptor subunit α; IL-13R α1, interleukin 13 receptor subunit α1; sIL-4R α, soluble IL-4R α; WT1, Wilms’ tumor 1; IL-10R α, interleukin 10 receptor subunit α; IL-10R β, interleukin 10 receptor subunit β; IL-6ST, interleukin 6 cytokine family signal transducer.

After cloning rat *Zhx3*, expression of all three ZHX proteins, ZHX1, ZHX2, and ZHX3, was noted in podocytes (7, 14, 15). Instead of a predominant nuclear expression, as noted in tubules (8) and most other organs, in vivo podocytes expressed these proteins predominantly at the podocyte cell membrane. ZHX proteins can form homodimers or heterodimers, both of which variably sequester the two nuclear localization signals (16–18). Based on confocal colocalization studies in rodent glomeruli, ZHX1 was predominantly present in the podocyte body, ZHX3 at the slit diaphragm, and ZHX2 at both locations (8) (Fig. 1). Co-immunoprecipitation studies using whole glomerular protein extracts showed the interaction of ZHX proteins with APA (podocyte body) and ephrin B1 (slit diaphragm) (8), suggesting that ZHX1-ZHX2 and ZHX2-ZHX3 heterodimers are tethered to the cytoplasmic domains of these proteins, respectively. Absence of APA or ephrin B1 or reduced expression of ZHX2 (BALB/cJ mice or *Zhx2^flox/flox^*-NPHS2 promoter *Cre^+/+^* mice) alters the proteinuria profile and suggests the presence of alternative transmembrane-binding partners (8), one of which is IL-4Rα (6), the receptor for IL-4 and IL-13 in podocytes. In proteinuric conditions, loss of heterodimerization of ZHX proteins induced migration of peripherally expressed proteins into the nucleus (7, 14). In the podocyte nucleus, one or more ZHX proteins regulate a vast majority of structurally and functionally important genes (7, 8). These include upregulation of *Angptl4*, a gene important in MCD, by ZHX1 and ZHX2 and its downregulation by ZHX3 (8), and downregulation of Wilms’ tumor 1 (WT1), a gene important in FSGS, by ZHX2 (7). In cultured human and mouse podocytes, nearly half of ZHX proteins are nuclear, making them less useful in studying the redistribution of ZHX proteins after podocyte injury (7). While conducting studies for human glomerular ZHX2 (8, 14), only one anti-ZHX2 antibody from Abnova reliably identified a single band at approximately 90 kDa on Western blot, and confocal imaging mirrored observations in rodents (14). Unfortunately, web-based resources like the Human Protein Atlas have characterized ZHX2 expression using low confidence or polyreactive antibodies (19).

While investigating human glomerular disease, low overall podocyte ZHX2 expression and increased nuclear ZHX1 were noted in MCD and low podocyte ZHX2 and increased nuclear ZHX2 and ZHX3 in FSGS (8). Based on the normal differential expression of ZHX proteins in the podocyte, MCD early pathways appear to originate in the podocyte body and FSGS in the slit diaphragm or foot process (Fig. 1). BALB/cJ mice are an interesting model of the *Zhx2* hypomorph state in which low *Zhx2* expression induced by a mouse endogenous retrovirus in intron 1 of *Zhx2* fails to suppress liver *Afp* in adult mice, resulting in very high plasma α-fetoprotein levels (20, 21). BALB/cJ mice develop more severe adriamycin nephrosis (FSGS model), and anti-APA antibodies induced *Angptl4* upregulation only in BALB/cJ mice, which also develop significantly higher albuminuria than BALB/c mice (8). Only BALB/cJ and *Zhx2^flox/flox^*-NPHS2 promoter *Cre^+/+^* mice develop albuminuria when injected with a common cold cytokine cocktail (model of MCD relapse) (6). In contrast, beyond a specific threshold of podocyte-specific *Zhx2* overexpression in a cell membrane distribution, NPHS2 promoter-*Zhx2* transgenic rats injected with adriamycin developed the collapsing variant of FSGS, with high podocyte nuclear ZHX2 expression (8).

### Genomic Defects in MCD

While exome sequencing did not find mutations in *ZHX2* in patients with primary glomerular disease (22, 23), insertions and deletions (InDels) were discovered far upstream of *ZHX2* in patients with MCD and FSGS (6). Some of these InDels were shared among patients, and replicating an insertion at Chr 8: 122,533,694 in a human podocyte line induced a *ZHX2* hypomorph state. Shared InDels tend to cluster at the beginning and end of rodent expressed gene *Slc22a22*, a prostaglandin transporter that is defunct in humans. Patients with collapsing glomerulopathy, including those after severe acute respiratory syndrome coronavirus 2 (SARS-CoV-2) infection, had different InDels, including some shared, and future studies will investigate if these InDels can induce *ZHX2* upregulation (6). By comparison, patients with diabetic nephropathy, the most common cause of nephrotic syndrome, did not have shared InDels upstream or far upstream of *ZHX2* (6). Interestingly, the only two shared InDels in patients with diabetic nephropathy were noted upstream of the neighboring gene *HAS2*, and this could have a potential role in the progression of tubulointerstitial fibrosis in diabetic nephropathy.

Unlike exome-based disease-causing gene variants that are often disease specific, shared intergenic region InDels that affect *ZHX2* expression in MCD and FSGS suggests a multitiered complex pathogenesis of *ZHX2*-mediated podocyte diseases. At least two factors beyond the *ZHX2* hypomorph state likely determine whether the disease evolves into MCD or FSGS. Differential distribution of ZHX proteins within the podocyte (discussed above in *ZHX Proteins and MCD*) and the co-occurrence of genomic defects in podocyte slit diaphragm and foot process protein genes in FSGS (24). Although most patients with FSGS do not have disease-causing gene variants based on exome sequencing, studies are needed to critically analyze intronic regions within and intergenic regions around these genes for the presence of InDels that modify gene expression. In our studies, at least half of the patients with known FSGS gene disease-causing variants also had InDels in *ZHX2* introns or upstream of *ZHX2* (6).

### Podocyte *ANGPTL4* Upregulation in MCD and the Initiation of Hyposialylation Injury

ANGPTL4 is a secreted glycoprotein with low constitutive expression in podocytes (9). After its discovery as a highly upregulated glomerular gene (9) in discovery phase studies (7), screening rat models of human glomerular disease revealed highest upregulation in a MCD model, where increased expression starts just before the onset of proteinuria, peaks a few days later, and declines before the end of proteinuria (9). Lesser transient upregulation after the onset of proteinuria occurred in membranous nephropathy (MN), and there were no changes in collapsing FSGS. Patients with MCD biopsied immediately after relapse had high podocyte staining for ANGPTL4 (9). A transgenic rat overexpressing rat ANGPTL4 specifically from podocytes developed most of the cardinal manifestations of MCD, albeit with a more gradual onset, including massive and selective proteinuria, diffuse foot process effacement, loss of glomerular basement membrane charge, and absence of tubulointerstitial fibrosis despite many months of proteinuria (9, 25). In addition, in vivo glucocorticoid sensitivity of the *Angptl4* gene was noted in a rat MCD model (9). Assessment of glomerular ANGPTL4 in rodent models revealed two interesting features: the ability to form oligomers and its migration specifically in MCD models at two isoelectric point (pI) clusters, high pI and neutral pI. In contrast, a MN model showed neutral pI glomerular ANGPTL4 only (unpublished observations). Using lectin binding, the neutral pI form was noted to have sialic acid (sialylated ANGPTL4), whereas the high pI form was deficient in sialic acid residues (hyposialylated ANGPTL4), and this phenomenon was replicated in a cultured podocyte cell line (9). Improving sialylation using *N*-acetyl-d-mannosamine (ManNAc), a precursor of sialic acid, increased the production of neutral pI protein and reduced proteinuria in podocyte-specific ANGPTL4 overexpressing transgenic rats and a rat MCD model (9). In human studies, this high pI ANGPTL4 was noted in the plasma of patients with MCD in relapse, but not in patients with MN, FSGS, or MCD in remission, and also in the urine of patients with MCD, and not in patients with FSGS (9).

**Figure 2.**
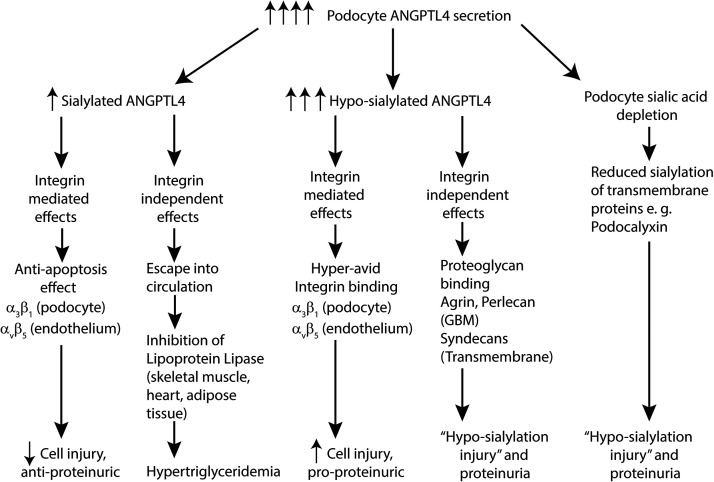
Flowsheet showing consequences of podocyte angiopoietin-like 4 (*ANGPTL4*) upregulation and the pathogenesis of “hyposialylation injury” in minimal change disease (MCD). GBM, glomerular basement membrane.

How does hyposialylated ANGPTL4 cause glomerular injury? Experimental MCD podocytes secrete a combination of sialylated and hyposialylated ANGPTL4 (9) (Fig. 2), based on available and recyclable sialic acid stores, both of which have local integrin-mediated effects in the glomerulus (10). ANGPTL4 binds to integrins β_1_ (podocyte α_3_β_1_) and β_5_ (glomerular endothelial α_v_β_5_) but not integrin β_3_ (glomerular endothelial α_v_β_3_) (26). The binding affinity of hyposialylated ANGPTL4 to integrin α_v_β_5_ (10) and integrin α_3_β_1_ (unpublished) is several-fold higher than sialylated ANGPTL4. In addition, hyposialylated ANGPTL4 secreted from podocytes binds avidly in an integrin-independent manner to cell membranes and the glomerular basement membranes, potentially through transmembrane (syndecans) and matrix (heparan sulfate) proteoglycans (9). Studies in a cultured glomerular endothelial cell-oxidative injury model prove that sialylated ANGPTL4 significantly reduces and hyposialyated ANGPTL4 significantly increases cell injury (10). Whether this increased cell injury is exclusively related to hyper-avid integrin binding or integrin-independent effects, or a combination of both, is not known. Because of more extensive glomerular binding of hyposialylated ANGPTL4, much of ANGPTL4 that escapes into the circulation via the efferent arteriole is neutral pI-sialylated protein (9). One must remain cognizant of the fact that a large amount of circulating sialylated ANGPTL4 is secreted from peripheral tissues (10) (see *Systemic Effects of Circulating ANGPTL4*).

There are likely additional intermediate and long-term effects of podocyte sialic acid depletion (Fig. 2). It is likely that hyposialylation-mediated injury initiated by massive ANGPTL4 upregulation is maintained by additional putative ZHX1-upregulated podocyte-secreted hyposialylated proteins, since *Angptl4* upregulation tends to subside in rat MCD models (9). The long-term sequelae of continued secretion of podocyte proteins would include reduced podocyte sialic acid stores and consequently reduced sialic acid incorporation into structural proteins like podocalyxin (27, 28), in which both sialic acid and sulfate residues contribute to charge (29). Sialylation-deficient proteins continue to be under intense investigation in our laboratory. In addition, urinary hyposialylated ANGPTL4 is under investigation as potential mechanistic biomarker for new onset MCD or MCD relapse (25).

### Loss of Glomerular Basement Membrane Charge

A common question in MCD is the link between the loss of glomerular basement membrane (GBM) charge and selective proteinuria. Podocyte-secreted ANGPTL4 is the only protein directly implicated in loss of glomerular GBM charge (9). However, several phenomena develop concurrently in podocyte *Angptl4* overexpressing transgenic rats: production of high pI hyposialylated ANGPTL4, selective proteinuria, loss of GBM charge, and effacement of foot processes (9). While they are all linked to hyposialylated ANGPTL4 production, they are not necessarily linked to each other. It is more likely that loss of GBM and cell surface charge in the early stages of disease is an epiphenomenon related to binding of hyposialylated high pI ANGPTL4 to matrix (agrin and perlecan) and transmembrane (syndecan) proteoglycans. As the disease persists, the additional factor of reduced podocyte sialic acid reserve and reduced incorporation of sialic acid into transmembrane proteins (e.g., podocalyxin) also contributes. To understand selective proteinuria, it is important to recognize that the two dominant proteins in plasma are albumin (around 70 kDa) and immunoglobulin (around 150–160 kDa). Proteins less than 25 kDa filter freely through the GBM and are reabsorbed in the proximal tubule. When the smaller dominant protein (albumin) accounts for the bulk of the proteinuria, this phenomenon is referred to as selective proteinuria. Given that MCD is more histologically uniform than either MN or FSGS, it is possible that the genomic and other factors that predispose to MCD are translated more uniformly than those in FSGS or MN. Thus, the more realistic explanation for selective proteinuria in MCD is in the uniformity or symmetry of the molecular and histological defect, compared with FSGS or MN, rather than a direct effect on GBM or cell surface charge. Much of the excitement behind podocalyxin charge in MCD subsided once it was recognized that the phenotype of podocyte podocalyxin knockout mice had no similarity to MCD (30) and that the interaction of podocalyxin with the actin cytoskeleton is mediated via ezrin and Na^+^/H^+^ exchange regulatory cofactor 1 (NHERFl) (31, 32).

### Systemic Effects of Circulating ANGPTL4

Circulating ANGPTL4 levels increase in all forms of nephrotic syndrome and are mostly secreted from adipose tissue, skeletal muscle, the heart, and the liver (10). This form of ANGPTL4 is well sialylated and nearly exclusively migrates in the neutral pI range. MCD podocytes secrete additional sialylated and some hyposialylated ANGPTL4 into the circulation (9), whereas MN is associated with mild podocyte ANGPTL4 secretion of the sialylated form (9, 10) (see also *Podocyte ANGTPTL4 Upregulation in MCD*). Peripheral organ secretion of circulating sialylated ANGPTL4 occurs at a stage when proteinuria crosses into the nephrotic threshold and is essentially a feedback mechanism to reduce proteinuria via the ANGPTL4 interaction with glomerular integrins (10, 33, 34). The overwhelming glomerular effects of hyposialylated ANGPTL4 and hyposialylation-mediated injury limit the efficacy of this feedback loop in MCD. Circulating ANGPTL4 also induces hypertriglyceridemia via lipoprotein lipase inhibition, and studies in rat models of diabetic nephropathy using injection of recombinant-mutated human ANGPTL4 have suggested that the antiproteinuric effect occurs at a lower dose than the hypertriglyceridemic effect (10). Recombinant mutated human ANGPTL4 is being developed as a potential therapeutic for chronic kidney disease (5). In addition, and unrelated to the ZHX-ANGPTL4 pathway, another recent study has shown that hypercholesterolemia of nephrotic syndrome is initiated by the secretion of PCSK9 from cortical collecting duct cells, where PCSK9 is a chaperone protein for the epithelial sodium channel (ENaC) (11). Therefore, hypertriglyceridemia in nephrotic syndrome appears to mechanistically correlate with proteinuria, and hypercholesterolemia with enhanced sodium absorption.

The absence of acute onset of edema, a key clinical feature of human MCD, in podocyte-specific ANGPTL4 overexpressing rats attests to the multigenic origin of human disease manifestations. Since the sudden development of edema requires both sodium retention and an increase in peripheral capillary endothelial permeability, it is likely that additional putative podocyte-secreted proteins (perhaps ZHX1 regulated) beyond ANGPTL4 are required for this effect.

### Circulating Proteins That Trigger MCD Relapse

Despite an interesting hypothesis put forward five decades ago (35), the purported T cell factor that induces MCD relapse remained elusive. We used a different approach by including some interesting features in the history and clinical profile of patients with MCD (6) (Fig. 1). Common colds, often caused by rhinoviruses, precede half of all event-defined episodes of relapse of steroid-dependent nephrotic syndrome or MCD (4). Also, half of the patients with MCD have some form of atopy (3). First, a list of cytokines released during a common cold, that could potentially leak into the circulation and have “distant” effects on glomerular cells, was assembled (6). Next, this list was trimmed based on the presence of receptors in the glomerulus to create a cytokine cocktail (6). Since chronic atopy can be associated with the upregulation of both transmembrane and a soluble spliced variant of IL-4Rα (36), IL-4Rα was added to the cocktail. Finally, since the rhinovirus receptor in nasal sinus epithelium, intercellular adhesion molecule-1 (ICAM-1), is often cleaved during acute infection and appears in the circulation, soluble ICAM-1 (sICAM-1) was added to the cocktail (6). After dose optimization, a common cold cytokine cocktail was developed to mimic a common cold cytokine storm, and at low doses induced albuminuria in *Zhx2* hypomorph BALB/cJ but not *Zhx2^+/+^* BALB/c mice. Equivalent doses of individual cocktail cytokines could not induce albuminuria in BALB/cJ mice (6). By subtracting individual cytokines from the cocktail and statistical comparison with the intact cocktail, major (TNFα, IL-4Rα, IL-6, and IFN-γ) and minor (IL-10, IL-2, and ICAM-1) contributors were identified. Since mice do not express IL-2Rα in podocytes, IL-2 may be a minor contributor in mice but could still be a major contributor in humans. The designation of ICAM-1 as a minor contributor opens the possibility of other common cold viruses that bind receptors other than ICAM-1 (i.e., viruses other than rhinovirus A and B) in inducing relapse on human glomerular disease. As a further proof of concept, albuminuria induced by common cold cocktail could be replicated in podocyte-specific *Zhx^flox/flox^*-NPHS2 promoter *Cre*^+/+^ mice, whereas *Zhx2^flox/flox^* control mice did not develop albuminuria (6). The most important concept that has crystallized from these studies is that, like the majority of biological processes, relapse of MCD is dependent on a combination or complex of soluble proteins (6) rather than single proteins, as has been postulated in the past (35). These proteins have diverse origins, e.g., TNF-α from macrophages, soluble IL-4Rα from epithelial cells and B cells, IFN-γ from T cells and natural killer (NK) cells, IL-6 from sites of acute or chronic inflammation, IL-10 from monocytes, and IL-2 from T cells.

Cytokine cocktail components target all three cells in the glomerular tuft, suggesting that a significant change in intercellular cross talk occurs during the development of MCD relapse (6) (Fig. 1). Among the three cocktail-relevant cytokine receptors expressed in human in vivo podocytes [tumor necrosis factor receptor type 1 (TNFR1), IL-2 receptor subunit α (IL-2Rα), and the IL-4 receptor α (IL4α)-IL-13 receptor α1 (IL-13Rα1) complex], *Il4Ra*^−/−^ mice in a *Zhx2* hypomorph background do not develop albuminuria induced by cytokine cocktail. This suggests a complex synergy at least between the IL-4Rα-IL-13Rα1 complex with TNFR1 in podocytes, since IL-2Rα is absent in mouse podocytes. Studies using CRISPR-modified *ZHX2* hypomorph-cultured human podocytes incubated with the human common cold cytokine cocktail show absence of the classic STAT6 pathway activation downstream of transmembrane IL-4Rα (6), since the cocktail lacks IL-4 and IL-13. Rather, the dominant role of transmembrane IL-4Rα in the common cold cytokine storm-induced relapse of MCD is related to its role as an alternative to APA in binding ZHX proteins (specifically ZHX1 in the podocyte cell body cell membrane) in the *Zhx2* hypomorph state. Albuminuria after injection of this cocktail in BALB/cJ mice is accompanied by the migration of ZHX1 from the cell membrane to the podocyte nucleus (6). Furthermore, like *Zhx2* hypomorph BALB/cJ mice, APA-deficient *Enpep*^−/−^ mice, which are *Zhx2^+/+^*, also develop albuminuria after injection of the common cold cytokine cocktail. In these studies with single and dual gene-deficient mice, the highest fold change in albuminuria from baseline after injecting this cocktail occurs in dual *Zhx2* hypomorph *Enpep*^−/−^ mice (6).

### New Generation Therapeutics for MCD

Current therapeutics using glucocorticoids, calcineurin inhibitors, or cytotoxic medications have been discussed in detail elsewhere (37). Before discussing different potential upcoming next-generation therapeutics for MCD, one must recognize that when using small animals as models, it is virtually impossible to replicate all aspects of MCD in a single model because of major physiological differences with humans (6). There are equally important genomic differences, as exemplified by the occurrence of a fully functional prostaglandin transporter gene *Slc22a22* in the *Has2*-*Zhx2* intergenic region in mice and rats, which is defunct in humans (6). Therefore, all aspects of therapeutics discussed here have been developed individually and could be implemented in a stepwise manner.

The ideal paradigm for treating future MCD would be as follows. The first episode of MCD (clinical profile and biopsy proven) would be treated with glucocorticoids that decrease hyposialylated ANGPTL4 production by reducing *ANGPTL4* upregulation (9). This would be followed by maintenance therapy with a sialyation-inducing agent to make relapses clinically mild, or even asymptomatic. Frequent symptomatic relapses, like those induced by a common cold, could be treated soon after the onset of the cold by depleting TNF-α or IL-6 or sIL-4Rα after confirming elevated plasma levels.

The fundamental treatment of hyposialylation-induced injury is to increase the sialic acid content in podocytes. The first compound investigated, ManNAc, significantly improved ANGPTL4 sialylation and reduced proteinuria in rats (9), but subsequent due diligence studies showed several-fold increases in *O*-GlcNAcylation of proteins (38). Since *O*-GlcNAcylation is a mediator of diabetes-related complications, long-term use of ManNAc as maintenance therapy, as would be required in patients with MCD, could potentially have diabetes-like side effects in the absence of diabetes. For this reason, we abandoned this compound several years ago and are developing other strategies to improve sialylation.

Recent rodent studies have suggested that the depletion of TNF-α, IL-6, and sIL-4Rα soon after the onset of a common cold could potentially prevent MCD relapse in patients who relapse frequently (6). Albuminuria induced by common cold cytokine storm cocktail in *Zhx2* hypomorph mice is reduced significantly by the depletion of TNF-α, IL-6, and sIL-4Rα 1 h after cocktail injection. This approach merits clinical trials, since anti-TNF-α (adalimumab, infliximab, golimumab, certolizumab, and others), anti-IL-6 (siltuximab), and anti-IL-4Rα (dupilumab) antibodies are currently in clinical use. The ideal patient for initial clinical trials would be an individual with a history of frequent relapse triggered by a common cold.

## OTHER PREVIOUSLY INVESTIGATED PATHWAYS

Numerous scientists have proposed or investigated other proteins or pathways to explain the pathogenesis of MCD. Unlike the ZHX-ANGPTL4 pathway (Table 1), none of these studies were designed or conducted to explain all the cardinal manifestations of MCD or its clinical associations. Some of these studies investigated single candidate circulating proteins that could induce proteinuria and partial foot process effacement, often after injecting large unphysiological doses or raising plasma levels over prolonged durations, but fell short of explaining specific features of MCD and disease associations discussed above (39–57). Others explored key structural podocyte proteins, either constitutively expressed (58) or inducible (59), as a way of explaining foot process effacement and proteinuria, but are likely to be peripheral pathways, since they lack specificity to MCD, and also do not explain specific disease features. Since the publication of mechanisms of MCD relapse induced by a common cold cytokine storm (6), two additional limitations of these past studies become apparent. First, none of these above studies was conducted in a *Zhx2* hypomorph background, so any link to the ZHX-ANGPTL4 pathway remains unexplored. Second, single cytokines injected into rodents at physiological or near physiological doses have minor effects on albuminuria compared with more robust albuminuria and histological changes induced by cytokine cocktails (6). Since biological processes that mediate glomerular disease are likely to be complex and alter cross talk among various cell types, the likelihood of a central role of single proteins explored in most of the studies discussed in the following paragraphs is remote.

### Shalhoub’s Hypothesis and T Lymphocyte Factor Studies

In a hypothesis proposed nearly five decades ago, Shalhoub (35) suggested that MCD was triggered by a circulating chemical mediator secreted by T cells. The justification for this hypothesis was at best superficial and included the following clinical associations: absence of a humoral antibody response (i.e., no involvement of B cells), remission induced by measles (modifies “cell-mediated immunity”), therapeutic effects of glucocorticoids and cyclophosphamide (improve “cell-mediated immunity”), and occurrence in Hodgkin lymphoma. Over time, the literature showed that glucocorticoid response elements are abundant in the human genome and are not limited to immune cells (60), the Reed-Sternberg cell in Hodgkin lymphoma is a modified B cell (61), and only about 1% of patients with Hodgkin lymphoma develop nephrotic syndrome (62). Despite these limitations, this hypothesis triggered a series of studies on proteins, “soluble factors,” and cytokines secreted from T cells, all of which made limited progress because all of the reasons listed in the prior paragraph. In some cases, proteins secreted from T cells or peripheral blood mononuclear cells isolated from patients with nephrotic syndrome were studied but did not adequately address whether these factors were a cause or effect of nephrotic syndrome. The most interesting among these studies investigated individually the effects of IL-4 or IL-13 (39, 40), two cytokines involved in the allergy pathway, on podocytes, since podocytes express the corresponding receptors and this ties into the association with atopy. However, in the IL-13 studies, albuminuria increased less than 10-fold in rats after 10 wk, and the levels of IL-13 attained were extremely high, thereby diluting relevance to clinical MCD (40). This study more closely mimicked a prolonged acute atopic cytokine profile, whereas our studies on common cold-induced MCD relapse combine the effects of chronic atopy (high sIL-4Ra levels) with the cytokine profile of an acute common cold (6). On a different note, high IL-13 (although not as high as the rat IL-13 study discussed here) and IL-4 levels are noted in intensive care unit-admitted patients with coronavirus disease 2019 (COVID-19), but not in patients with a common cold, and IL-13 and IL-4 are included in COVID-19 cytokine storm cocktails in our studies (6).

### Hemopexin

This is an interesting nonimmune cell-secreted protein investigated for a potential role as a “soluble factor” that induces MCD (54–56). Hemopexin binds to heme, and plasma levels are increased in patients with MCD. In vivo, hemopexin also binds sialoglycoproteins in podocytes and triggers transient mild proteinuria and foot process effacement in perfused rats. Many of the effects of hemopexin in glomeruli have been so far attributed to its protease activity (57). However, none of the hemopexin studies was conducted in a *Zhx2* hypomorph state, which could potentially amplify all of the above effects and could also potentially trigger migration of ZHX proteins in podocytes from the cell membrane into the nucleus. Further investigation of this phenomenon is warranted, since proteins other than the IL-4Rα likely also serve as alternative transmembrane-binding proteins for ZHX1 in the *Zhx2* hypomorph state.

### CD80

CD80/B7.1 is a T cell costimulatory molecule that is induced in a wide variety of inflammatory and noninflammatory glomerular conditions (59, 63). It has been investigated as a mechanism for foot process effacement development and proteinuria but not as a specific mechanism for MCD. Some studies have suggested urinary CD80 as a screening biomarker for MCD (64). The mechanism for such specificity, if it is indeed true, remains unresolved. The CD80 pathway is at best a peripheral pathway in MCD due to induction in a wide variety of glomerular diseases, and absence of constitutive expression in podocytes makes it an unlikely candidate as the first event involved in the trigger of acute relapse.

### c-Maf-inducing Protein

c-Maf-inducing protein (C-MIP) is upregulated in T cells in patients with MCD, increased in podocytes during MCD relapse, and repressed during remission (65). However, the upregulation in podocytes in patients with FSGS and MN argues against this pathway as having a primary role in MCD pathogenesis (58, 66, 67) and has not been investigated in detail in that context either. It is more likely to be involved in a peripheral pathway in the pathogenesis of proteinuria or foot process effacement or both.

### CD20 and the Rituximab Effect

The partial efficacy of the anti-CD20 antibody drug rituximab in patients with nephrotic syndrome has raised the potential role of B cell depletion in the treatment of various nephrotic states (68). However, there is no known role of IgG, the major secretory product of B cells, in MCD. Another study showed that rituximab binds also to podocyte sphingomyelin phosphodiesterase acid like 3B, thereby suggesting that its antiproteinuric effect may be independent of B cell depletion (69). Moreover, some patients maintain prolonged remission after rituximab infusion despite reconstitution of B cells (70). The mechanism of this beneficial rituximab effect remains unresolved and is unlikely to be CD20 related. There is unlikely to be a significant role of CD20 in MCD pathogenesis.

### Anti-Nephrin Antibodies and MCD

A recent study (71) has shown the presence of anti-nephrin antibodies in the circulation of nearly a quarter of patients with MCD and suggested a causal role of these antibodies in MCD pathogenesis. A primary role of anti-nephrin antibodies in MCD pathogenesis is unlikely for several reasons. Of two prior major experimental studies in rats using anti-nephrin antibodies (72, 73), one caused very mild proteinuria (73), and neither had associated foot process effacement. Histology in this study (71) relies heavily on colocalization of nephrin with endocytosed IgG in podocytes, implying that specific anti-nephrin antibodies are endocytosed. Since podocytes actively endocytose glycoproteins and IgG is one of the most abundant plasma glycoproteins, it is challenging to accept this argument. The occurrence of anti-nephrin antibodies in this cohort is nevertheless interesting and raise the possibility of an immune response to nephrin that is hyposialylated (like podocalyxin discussed earlier) after the nephrotic state and hyposialylation injury are established. Proof for this concept will require epitope mapping of these circulating anti-nephrin antibodies using both sialylated and hyposialylated nephrin. If these epitopes are restricted to sites of glycosylation, one could consider the possibility of these antibodies modifying the clinical course of podocyte cell body-based MCD mechanisms in a *ZHX2* hypomorph state with slit diaphragm-related signals seen classically with FSGS pathophysiology (Fig. 1, *bottom* and *middle*). This could also explain the progression of histological MCD to histological FSGS and end-stage renal disease in the index case discussed in this study (71).

## GRANTS

This work is supported by National Institutes of Health grants R01DK128203, R01DK129522, R01DK109713, and R01DK111102 (to S.S.C.) and K01DK096127 and R01DK126926 (to L.C.C.).

## DISCLOSURES

S.S.C. is founder and president of GDTHERAPY LLC. S.S.C. is inventor on the following patents: United States 14/943,167 and United States 15/803,524 for sialylation-based therapeutics of kidney disease; PCT/US2011/039255 and PCT/US2014/030009 for recombinant-mutated human ANGPTL4-related treatment of proteinuria and chronic kidney disease; PCT/US2019/042748 for therapeutic depletion of specific cytokines to prevent common cold-induced relapse or worsening of human glomerular disease; PCT/US2022/47254 for therapeutic depletion of cytokine combinations to ameliorate systemic manifestations and reduce mortality in severe viral cytokine storms; PCT/US2022/47263 for therapeutic reduction of ZHX2 expression to reduce morbidity and mortality from cytokine storms; and PCT/US2023/062503 to use recombinant-mutated human ANGPTL4 to treat multisystem disease resulting from cytokine storms. None of the other authors has any conflicts of interest, financial or otherwise, to disclose.

## AUTHOR CONTRIBUTIONS

S.S.C. and L.C.C. prepared figures; S.S.C. and L.C.C. drafted manuscript; S.S.C. and L.C.C. edited and revised manuscript; S.S.C. and L.C.C. approved final version of manuscript.
